# Draft Genome Sequence of Methylobacterium sp. Strain V23, Isolated from Accretion Ice of the Antarctic Subglacial Lake Vostok

**DOI:** 10.1128/genomeA.00145-18

**Published:** 2018-03-08

**Authors:** Amanda Sapp, José C. Huguet-Tapia, Maximiliano Sánchez-Lamas, Giuliano T. Antelo, Emiliano D. Primo, Jimena Rinaldi, Sebastián Klinke, Fernando A. Goldbaum, Hernán R. Bonomi, Brent C. Christner, Lisandro H. Otero

**Affiliations:** a Department of Microbiology and Cell Science, University of Florida, Gainesville, Florida, USA; b Department of Plant Pathology, University of Florida, Gainesville, Florida, USA; c Fundación Instituto Leloir, IIBBA-CONICET, Buenos Aires, Argentina; d Departamento de Biología Molecular, FCEFQyN, Universidad Nacional de Río Cuarto, Río Cuarto, Córdoba, Argentina; e Plataforma Argentina de Biología Estructural y Metabolómica PLABEM, Buenos Aires, Argentina; f Centre de Biochimie Structurale, Université de Montpellier, Montpellier, France

## Abstract

Here, we report the draft genome sequence of Methylobacterium sp. strain V23, a bacterium isolated from accretion ice of the subglacial Lake Vostok (3,592 meters below the surface). This genome makes possible the study of ancient and psychrophilic genes and proteins from a subglacial environment isolated from the surface for at least 15 million years.

## GENOME ANNOUNCEMENT

Lake Vostok, the largest subglacial lake in Antarctica, is covered by ∼4 km of glacial ice up to 420,000 years old and has been isolated from direct interaction with the atmosphere for at least 15 million years (1, 2). As such, the extreme conditions and isolation of Lake Vostok make it a unique environmental source of ancient eukaryotic and prokaryotic microorganisms with adaptations to low temperature.

Christner et al. (3) recovered bacterial isolates from melted samples of the Vostok ice core that originated from the zone of accretion ice at a depth of 3,592 m below the surface. Identification of the isolates based on small-subunit rRNA gene sequencing revealed that members of the genera Brachybacteria, Methylobacterium, Paenibacillus, and Sphingomonas retained their viability in subglacial Lake Vostok. Here, we report the draft genome sequence of Methylobacterium sp. strain V23.

Isolate V23 was cultured as previously reported (3), and its genomic DNA was isolated using the UltraClean microbial DNA isolation kit (Mo Bio, Inc., USA). Whole-genome *de novo* sequencing was performed with 100-bp paired-end reads using the Illumina HiSeq 4000 platform (Macrogen, Inc.). The DNA library was prepared using a TruSeq DNA PCR-free kit according to the instructions in the TruSeq DNA PCR-free sample preparation guide (Macrogen, Inc.). A total of 21,879,238 paired-end reads (average coverage of 400×) were assembled with SPAdes version 3.10 (4) into 138 contigs with an *N*_50_ contig size of 176,075 bp. Assembled contigs were corrected by remapping the raw reads with Bowtie 2 (5) and Pilon (6).

Gene prediction and annotation were performed using the Rapid Annotations using Subsystems Technology (RAST) annotation server version 2.0 (7). Additional annotation was conducted using the NCBI Prokaryotic Genome Annotation Pipeline (https://www.ncbi.nlm.nih.gov/genome/annotation_prok). The analysis of predicted genes and products was performed using the SEED viewer version 2.0 (8). The genome assembly was 5,935,666 bp in length with a G+C content of 68.1%. The RAST annotation server predicted 5,535 protein-coding sequences (CDSs) and 48 tRNAs. A total of 2,131 CDSs (39%) were assigned to subsystems (collections of functionally related protein families), while 3,404 (61%) remained unassigned. A total of 440 subsystems were identified, with most of the genes involved in (i) carbohydrate metabolism (352 genes); (ii) amino acids and derivatives (327); (iii) cofactors, vitamins, prosthetic groups, and pigments (299); and (iv) protein metabolism (270).

A whole-genome comparison from Methylobacterium sp. strain V23 indicated that the most closely related sequenced genome corresponds to the bacterium Methylobacterium sp. Leaf125 (European Nucleotide Archive number GCA_001423085.1), isolated from Arabidopsis thaliana leaves (9). The average nucleotide identity is 97% between these strains and less than 85% in comparison with other members from the genus Methylobacterium. Interestingly, Methylobacterium sp. strain V23 is predicted to encode genes related to plant growth regulators, low temperature, sporulation, resistance to antibiotics, and photoreceptors, among others.

Thus, the availability of the Methylobacterium sp. strain V23 genome will be useful for further investigations into ancient and psychrophilic genes and proteins that may have relevance to evolutionary biology and biotechnology.

### Accession number(s).

The whole-genome shotgun project presented here has been deposited at GenBank under the accession number PDHT00000000.
